# Systematic evaluation of whole exome sequencing for copy number variation detection in prenatal diagnosis

**DOI:** 10.1002/ctm2.70761

**Published:** 2026-09-08

**Authors:** Lihua Yu, Rong Hu, Xingwang Wang, Yongchu Liu, Haowen Tan, Haoyu Dou, Yi Kong, Jian Lu, Hongke Ding, Yimo Zeng, Cuize Yao, Li'ai Pan, Shuqi Chen, Yan Zhang, Aihua Yin

**Affiliations:** ^1^ Medical Genetic Center Guangdong Women and Children Hospital Guangzhou China; ^2^ Medical Genetic Center Women and Children's Hospital Southern University of Science and Technology Guangzhou China; ^3^ Guangzhou Key Laboratory of Prenatal Screening and Prenatal Diagnosis Guangzhou China; ^4^ Maternal and Children Metabolic‐Genetic Key Laboratory Guangzhou China; ^5^ Department of Bioinformatics Aegicare (Shenzhen) Technology Co., Ltd. Shenzhen China; ^6^ Department of Medical Genetics SynerGene Education, Hejun College Huichang China; ^7^ Graduate School Guangzhou Medical University Guangzhou China

Dear Editor,

Whole exome sequencing (WES) showed high concordance (97.03%) with chromosomal microarray analysis (CMA) for prenatal copy number variation (CNV) detection in our large clinical cohort.^1^, ^2^ We further established a novel quality control (QC) framework based on Standard Deviation (STD) and Normalised Exon Depth Deviation Index (NEDDI) to standardise WES‐based CNV calling. Our large‐scale clinical validation provides robust empirical evidence for WES as a first‐line prenatal genetic diagnostic tool,^3^ addressing the limitations of sequential CMA+WES testing workflows in time‐sensitive prenatal settings.

In this retrospective study, 3426 families comprising 3529 fetuses/probands underwent parallel CMA and WES at Guangdong Women and Children Hospital (S1,S4). CNVs were classified as large (deletions ≥ 100 kb or duplications ≥ 500 kb) or small (deletions < 100 kb or duplications < 500 kb). Using simulated datasets generated from blended high‐ and low‐quality samples, we identified STD and NEDDI as the most reliable QC metrics for CNV calling accuracy (S4). Two quadratic decision boundaries were then established for large CNVs (NEDDI = 2.927 – 16.956 × STD + 25.243 × STD^2^) and small CNVs (NEDDI = 1.115 – 6.319 × STD + 9.701 × STD^2^) (S1).

We further validated this QC framework in an independent cohort of 500 CMA‐confirmed clinical samples (S3,S4), and this QC framework showed strong performance.^4^ For large‐CNV QC, sensitivity was 96.6%, the false‐positive rate (FPR) was 10.3%, and the negative predictive value (NPV) was 84.6% (S3,S5). For small‐CNV QC, sensitivity was 96.2%, the FPR was 23.8%, and the NPV was 99.1% (S3,S5). Validation across xGen and NEXome XP capture kits, as well as across MGISEQ‐T7 and GeneMind SURFSeq 5000 platforms, supported the transferability of the boundaries under local NEDDI normalisation (S1,S5).

Using a predefined segment‐level reciprocal overlap threshold of 50%, WES exhibited high concordance with CMA (the gold standard) for prenatal CNV detection.^1^, ^2^, ^5^ Concordance was 97.03% for pathogenic/likely pathogenic (P/LP) CNVs (196/202), 95.83% for mosaic cases (23/24), and 93.22% for regions of homozygosity (ROH, 55/59) (Table 1, S4).^6^ For P/LP CNVs, deletion concordance was 96.58% (141/146) and duplication concordance 98.21% (55/56) (S4). The six discordant cases all involved shared key pathogenic genes (e.g., *EXT1*) despite differing fragment sizes, due to WES's exonic focus and CMA's genome‐wide SNP probe coverage. The single mosaic aneuploidy discordance was attributed to confined placental mosaicism rather than inadequate WES sensitivity, and all mosaic P/LP deletions/duplications showed 100% concordance. In concordant ROH cases, WES identified 9 cases of uniparental disomy (UPD) and 1 mosaic UPD, with 4 cases harbouring homozygous P/LP SNVs in ROH regions (S4).

**TABLE 1:** **Consistency between CMA and WES in detecting CNVs, mosaicisms and ROHs**.

Subgroups		CMA detected CNVs	WES detected CNVs With ≥ 50% reciprocal overlap	Concordance rate (95% CI)
P/LP CNVs	Deletions	146	141	96.58% (92.19% to 98.88%)
	Duplications	56	55	98.21% (90.45% to 99.95%)
	**Total**	**202**	**196** ^†^	**97.03%** (93.65% to 98.90%)
Mosaic	Aneuploidy	12	11^‡^	91.67% (61.52% to 99.79%)
	P/LP deletions	3	3	100% (29.24% to 100%)
	P/LP duplications	9	9	100% (66.37% to 100%)
	**Total**	**24**	**23**	**95.83%** (78.88% to 99.89%)
ROH	≤ 3 ROHs in one case	59	55^§^	**93.22%**
	Mosaic uniparental disomy	1	1	100%

Abbreviations: CI, confidence interval; P/LP, pathogenic/likely pathogenic.

^†^ The six cases were classified as non‐concordant because they did not meet the predefined reciprocal overlap threshold (≥50%), despite agreement at the clinically relevant gene level.

^‡^ The discordance in one sample (Case No. CW2676) was attributed to biological heterogeneity between chorionic villi and amniocytes.

^§^ Among four discordant ROHs, three discordances were attributed to < 50% reciprocal overlap threshold with CMA results; one case lacked capture probes.

Notably, among 3274 CMA‐negative cases, WES detected 18 additional reportable small CNVs (S4), ranging in size from 111 bp to 89 kb, including exon‐level deletions in *SMN1* and duplications in *DMD*, and an 89 kb homozygous deletion involving *NPHP1*.^7^ All of these additional CNVs were validated by orthogonal methods (S3,S5). WES also resolved compound pathogenic variants (CNVs+SNVs/indels) in four recessive disorder cases, illustrating the advantage of simultaneous CNV/SNV analysis in a single assay. In addition, trio‐WES analysis showed that 72.58% (90/124) of CNVs classified as variants of uncertain significance (VUS) were inherited, thereby reducing clinical reporting ambiguity (S4).

Overall, WES's positive predictive value (PPV) for 223 CNVs was 98.65% (220/223), with 100% PPV for large deletions/duplications and < 500 kb duplications (Table 2). The remaining three were classified as false positives, and were all small exon‐level deletions (50 bp to 8.9 kb) most consistent with intra‐batch reference bias, local hybridisation artefacts, or probe‐capture blind spots. CMA‐detected P/LP CNVs showed a genome‐wide distribution with hotspots at 22q11.21, 15q11.2, 17q12 and 16p11.2 accounting for 34.65% (70/202) of variants (S1).

**TABLE 2:** Validation of CNVs detected by WES.

	CNVs detected by WES	Validated CNVs by other methods	PPV (95% CI)
Deletions ≥ 100 kb	143	143	100% (97.45% to 100%)
Deletions < 100 kb	23	20	86.96% (66.41% to 97.22%)
Duplications ≥ 500 kb	54	54	100% (93.40% to 100%)
Duplications < 500 kb	3	3	100% (29.24% to 100%)
**Total**	**223**	**220**	**98.65% (96.12% to 99.72%)**

Abbreviations: CI, confidence interval; PPV, positive predictive value.

Based on our local cost accounting, compared with a sequential CMA + WES workflow, an integrated WES‐based single test was estimated to reduce total testing costs by approximately 30%, while shortening turnaround time from 6–8 weeks to 3 weeks.^8^ Despite these advances, several limitations should be acknowledged, including the single‐centre design, the need for local QC calibration, WES's limited coverage of non‐coding regions (partially mitigated by targeted probes), and residual VUS interpretation challenges—substantially alleviated by trio analysis.

This study provides a validated QC framework for WES‐based prenatal CNV detection, and demonstrates that WES can achieve high concordance with CMA while offering clear advantages in detecting small intragenic CNVs and integrating CNV/SNV analysis to resolve compound pathogenic variants. Moreover, trio‐WES reduces VUS ambiguity via inheritance tracking. These findings support WES as a robust, efficient first‐line alternative to CMA for comprehensive prenatal genetic evaluation, that optimises clinical workflows, shortens diagnostic time, improves diagnostic yield, and addresses the unmet needs of monogenic disorder screening, supporting its adoption as a unified first‐line test in precision prenatal medicine.^9^, ^10^

We confirm that all original study data (including samples, sequencing protocols, validation methods, and supplementary materials) are retained in full, and the manuscript has undergone plagiarism checking with a similarity rate of < 5%. All authors have reviewed and approved this letter, and declare no conflicts of interest except Yongchu Liu and Haoyu Dou (employees of Aegicare (Shenzhen) Technology Co., Ltd.). The full original manuscript with all Figures  and tables is submitted as a single accompanying file.

## AUTHOR CONTRIBUTIONS

Conceptualization, Aihua Yin, Yan Zhang; Data curation, Lihua Yu, Rong Hu, Xingwang Wang, Haoyu Dou, Hongke Ding, Jian Lu; Formal analysis, Xingwang Wang, Haowen Tan, Haoyu Dou; Funding acquisition, Aihua Yin, Yimo Zeng; Supervision, Aihua Yin, Yan Zhang; Validation, Lihua Yu, Yi Kong, Yan Zhang, Cuize Yao, Li'ai Pan, Shuqi Chen; Visualization, Lihua Yu, Rong Hu, Xingwang Wang, Yongchu Liu, Haoyu Dou; Writing – original draft, Lihua Yu, Rong Hu, Xingwang Wang, Yongchu Liu, Haowen Tan, Haoyu Dou, Yimo Zhang; Writing – review & editing, All authors.

## ETHICS APPROVAL AND CONSENT TO PARTICIPATE

This study was approved by the Guangdong Women and Children Hospital Medical Ethics Committee (Approval Number: 20251014). Informed consent was obtained from all participants. For minors, incapacitated individuals, and prenatal cases, consent was provided by their parents or guardians.

## FUNDING INFORMATION

This work was supported by the National Key Research and Development Project (grant number 2024YFC2707001 to Y.Zhang), the National Nature Science Foundation of China (grant number 82171856 to A.Y.), and Guangdong Joint Research Fund (grant number 2022A1515220097 to Y.Zhang).

## Supporting information



Supporting Information

Supporting Information

Supporting Information

Supporting Information

Supporting Information
