# Supplementary material for: Systematic evaluation of whole exome sequencing for copy number variation detection in prenatal diagnosis
Source: Clin Transl Med. 2026 Sep 8;16(9):e70761. doi: 10.1002/ctm2.70761 (PMC13554711; doi:10.1002/ctm2.70761)
Supplement: Supplementary file 1 [file CTM2-16-e70761-s003.pdf]

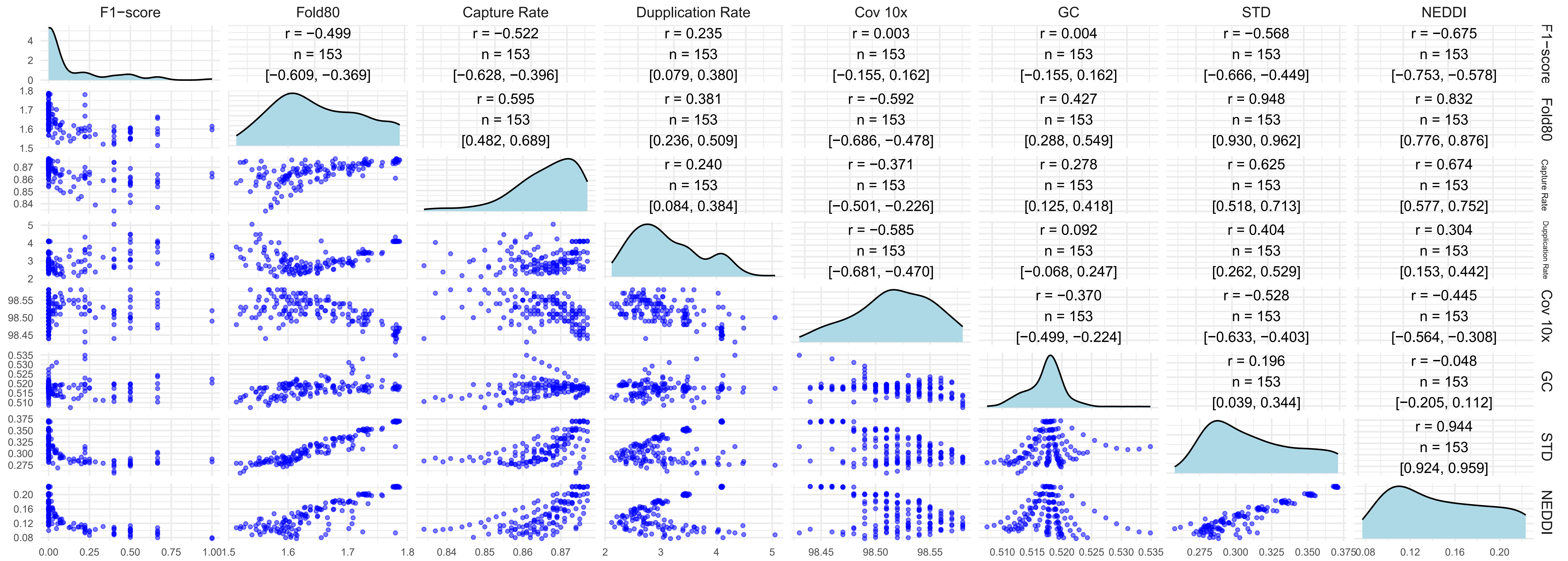

Fig. S1 Correlation analysis of QC metrics and CNV detection performance. Pairwise comparisons between quality control (QC) metrics and CNV detection performance indicators are shown for the simulated samples. Scatter plots in the lower triangle illustrate the distribution relationships between variable pairs, with each point representing an individual sample. Density plots on the diagonal panels depict the distribution characteristics of individual metrics. The upper triangle reports Pearson correlation coefficients with 95% confidence intervals in the format r (95% CI: [lower, upper]). Notably, the F1-score shows stronger negative correlations with NEDDI (r = -0.675; 95% CI: [-0.753, -0.578]) and STD (r = -0.568; 95% CI: [-0.666, -0.449]) than with Fold-80 base penalty (r = -0.499), indicating that NEDDI and STD more directly reflect CNV detection reliability and outperform traditional sequencing quality indicators in identifying samples prone to false positives or reduced detection sensitivity.

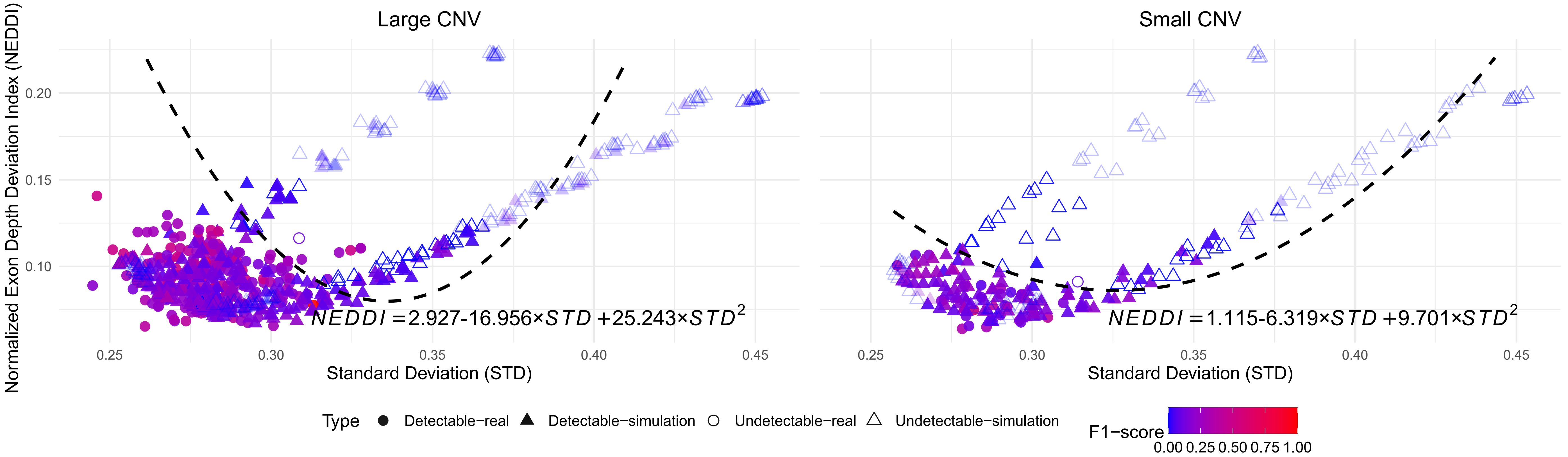

Fig. S2 Validation of QC thresholds. The proposed QC framework was evaluated on 500 CMA-confirmed clinical samples. Of the 204 samples (178 with only large CNVs, 25 with only small CNVs, and one with both) carrying CMA-positive P/LP CNVs, 96.6% (173/179) of samples with large CNVs met the QC thresholds, and 96.2% (25/26) of samples with small CNVs satisfied the QC thresholds. This supports the clinical applicability and robustness of the proposed quality control approach.

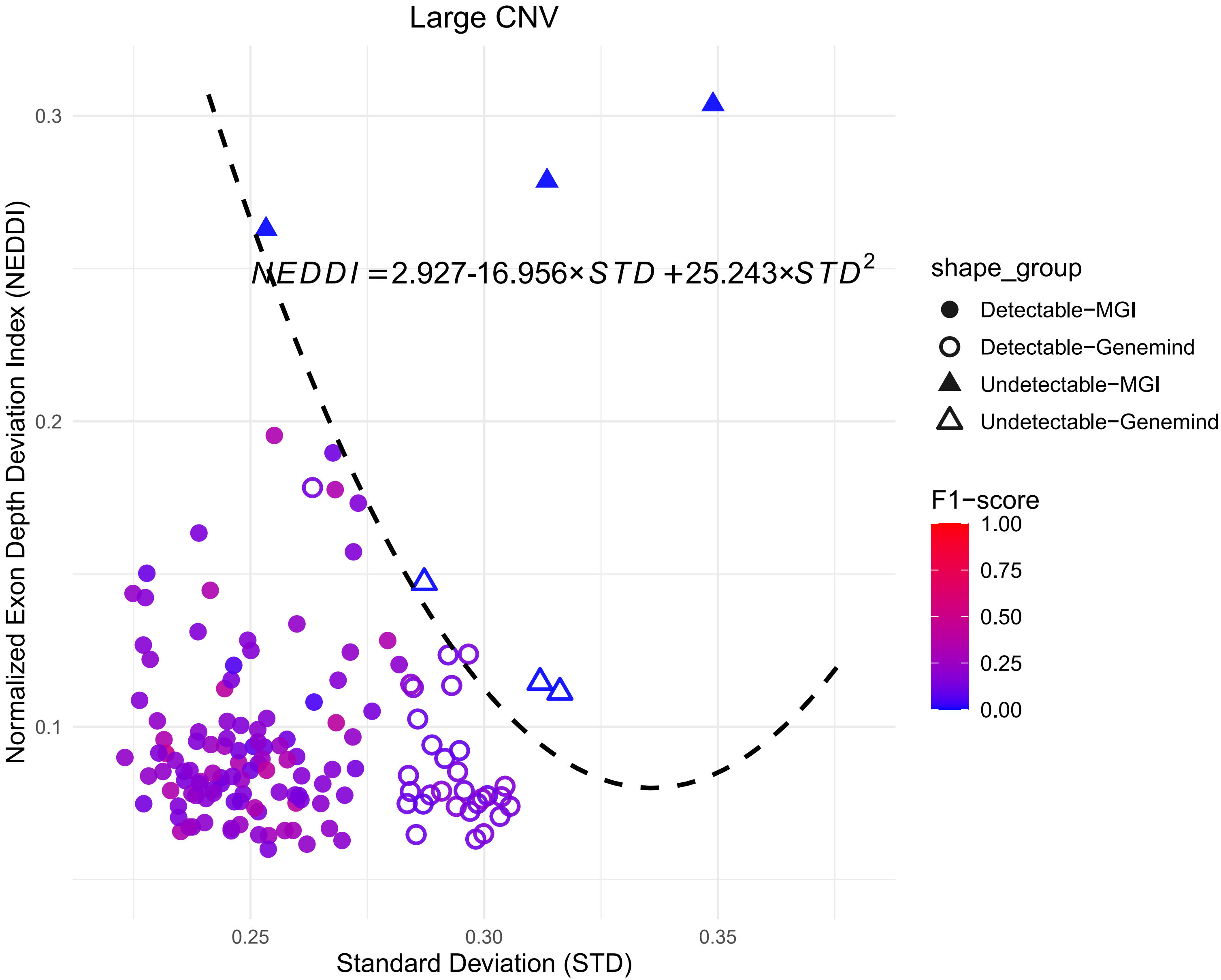

Fig. S3 Cross-platform validation of the large-CNV QC boundary. The proposed quadratic boundary for large CNVs was evaluated on 145 NEXome-captured samples across two independent platforms, comprising 112 MGI-T7 and 33 GeneMind SURFSeq 5000 samples. NEDDI was recomputed using each platform's cohort median. Of these, 95.2% (138/145) of samples fell below the boundary and were classified as Detectable, while 4.8% (7/145) of samples fell above and were classified as Undetectable. This demonstrates the direct portability of the QC threshold across sequencing platforms.

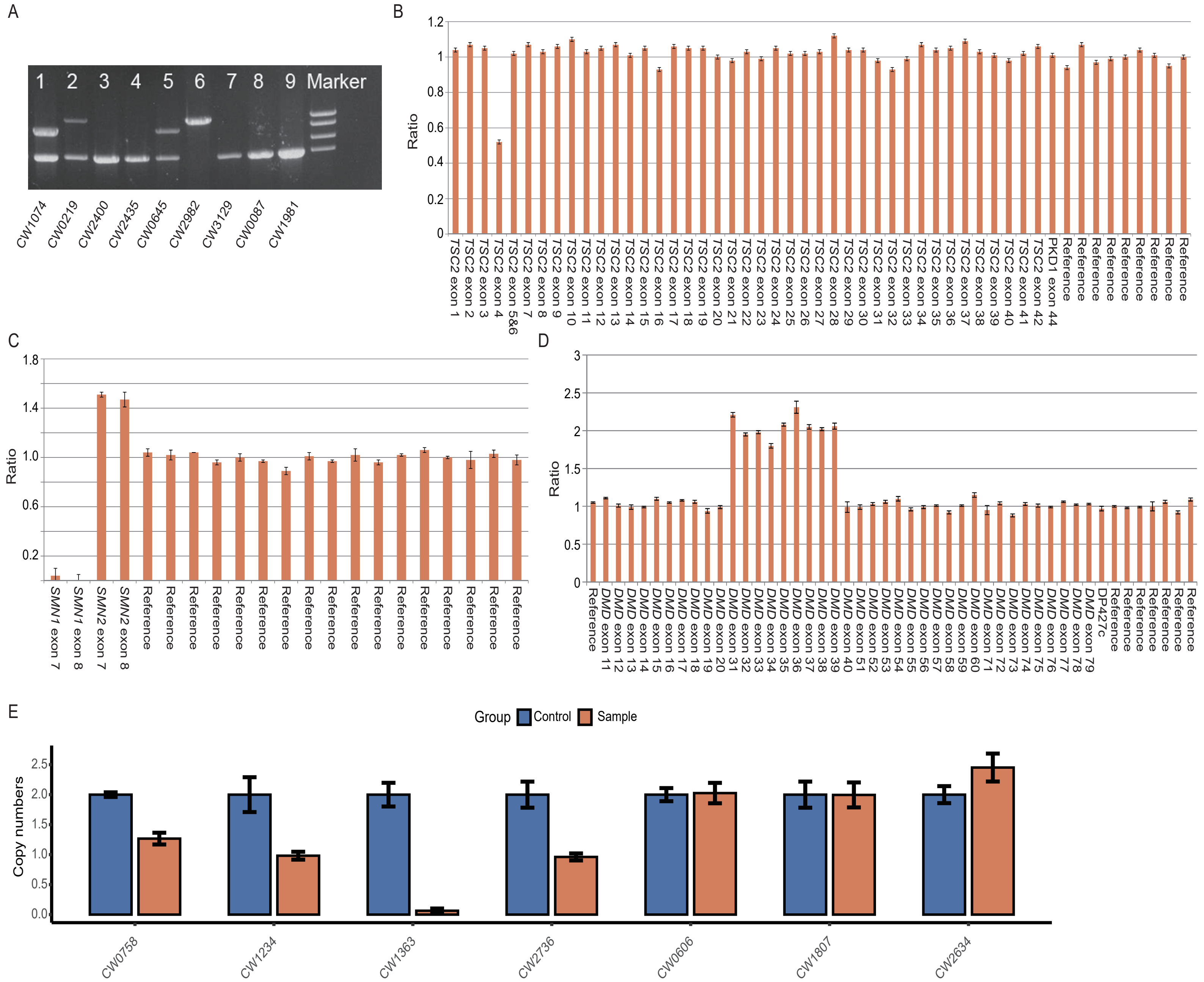

Fig. S4 WES-derived small CNVs validation by Gap-PCR, MLPA, and qPCR. (A) Gap-PCR validation of common α-thalassemia deletions. Marker lanes show bands for 3.7 kb deletion, normal allele, 4.2 kb deletion, and Southeast Asian ($--^{SEA}$) deletion. Among 9 cases: CW1074 ($--^{SEA}/\alpha^{WS}\alpha$), CW0645 ($--^{SEA}/\alpha^{WS}\alpha$), and CW0219 ($--^{SEA}/-\alpha^{3.7}$) exhibited compound heterozygous patterns, with the $\alpha^{WS}\alpha$ alleles confirmed by sequencing; CW2400, CW2435, CW3129, CW0087, and CW1981 showed homozygous $--^{SEA}$ deletions; CW2982 displayed homozygous $-\alpha^{3.7}$ deletions. (B-D) MLPA validation of exon-level deletions in *TSC2*, *SMN1*, and *DMD*. Case CW1054 (heterozygous deletion of *TSC2* exon 4) indicated tuberous sclerosis (B); CW0048 (homozygous deletion of *SMN1* exons 7-8) confirmed spinal muscular atrophy (SMA) (C); CW2311 (heterozygous duplication of *DMD* exons 31-39) required further clinical interpretation (D). (E) qPCR confirmation of WES-identified CNVs <100 kb. qPCR analysis of cases CW0758, CW1324, CW1363, and CW2736 revealed copy numbers (CN) of 1, 1, 0, and 1, respectively, fully concordant with WES-derived CNV calls. Conversely, three cases (CW0606, CW1807, and CW2634) showed normal diploid copy numbers (CN=2) by qPCR, confirming them as false-positive CNV detections by WES.
