# Supplementary material for: Systematic evaluation of whole exome sequencing for copy number variation detection in prenatal diagnosis
Source: Clin Transl Med. 2026 Sep 8;16(9):e70761. doi: 10.1002/ctm2.70761 (PMC13554711; doi:10.1002/ctm2.70761)
Supplement: Supplementary file 2 [file CTM2-16-e70761-s001.docx]

**Supplementary Methods**

**1 Quality control metrics for CNV detection in exome sequencing data**

**Standard deviation (STD)**

This metric quantifies intra-sample depth variability by evaluating exon-level sequencing depth. For each probe-targeted exon, the mean depth per segment is calculated. A frequency density plot is then generated to analyze depth distribution, from which the standard deviation (STD) of depth values across all exons is derived. STD, which reflects the heterogeneity of sequencing depth within a sample, is calculated as follows:

$$STD = \sqrt{\frac{1}{N}\sum_{i=1}^{N} \left( D_{i}- \bar{D} \right)^{2}}\div\bar{D}$$

N: Total number of exons in the sample,

$D_{i}$: Sequencing depth of the i-th exon,

$\bar{D}$: Mean sequencing depth across all exons in the sample.

**Normalized exon depth deviation index (NEDDI)**

This metric evaluates inter-sample depth consistency by comparing exon-level sequencing depths to population-wide median values. For each exon, the absolute difference between the target sample’s depth ($D_{i}$) and population median depth (M) is calculated. To ensure meaningful comparisons, the population median should be derived from reference samples with similar sequencing depth. Specifically, if the target sample's average depth is 100×, the reference set should include samples with depths within a ±20× range (i.e., between 80× and 120×), and preferably within a narrower window (i.e., between 90×and 110×). The mean of these absolute differences is then normalized by the sample’s mean depth ($\bar{D}$), yielding the normalized exon depth deviation index (NEDDI). This metric quantifies how uniform a sample’s exon depth is aligned with the expected population profile, defined as:

$$NEDDI =\frac{1}{N}\sum_{i=1}^{N} \left| D_{i}-M \right|\div\bar{D}$$

N: Total number of exons in the sample,

$D_{i}$: Sequencing depth of the i-th exon in the target sample,

M: Population-wide median sequencing depth for the i-th exon,

$\bar{D}$: Mean sequencing depth of the target sample.

**2 Derivation and validation of the STD–NEDDI quadratic QC boundaries**

**Fitting method**

To derive the quality-control (QC) boundaries for CNV detection, we fitted separate polynomial logistic regression models for large and small CNVs using STD, NEDDI, and STD² as covariates. Model fitting was performed by maximum likelihood estimation with negligible L2 regularization. To ensure adequate representation of the minority fail class, which comprised only 13–22% of the training data, we weighted fail samples according to their inverse class frequency during optimization. This prevents an overly permissive boundary that would allow poor-quality samples to slip through.

The quadratic decision boundary is derived analytically from the fitted logistic coefficients. The model predicts the probability of Pass as:

p(Pass) = σ(β₀ + β₁·STD + β₂×NEDDI + β₃×STD²)

Setting the logit to zero (p = 0.5) and solving for NEDDI yields the closed-form quadratic boundary:

NEDDI = (−β₀/β₂) + (−β₁/β₂)×STD + (−β₃/β₂)×STD²

**Optimization objective**

The optimization objective was to maximize specificity while maintaining sensitivity ≥ 80%, reflecting the clinical priority of preventing poor-quality samples from passing QC. To achieve this objective, we calibrate the decision threshold by searching downward from the base p = 0.5 model. We search p cutoffs from 0.01 to 0.50 in 0.01 increments. A threshold p < 0.5 shifts the intercept of the quadratic boundary downward to a lower NEDDI cutoff, making the QC more conservative. Among the candidate p cutoffs, we selected the threshold that achieved the highest specificity while maintaining sensitivity ≥ 80%. If multiple thresholds satisfied this criterion, the one with the highest specificity was retained.

A key methodological refinement is that large mosaic duplication samples are excluded from the large boundary fitting. Mosaic CNVs have inherently lower detection sensitivity; a subset of mosaic fail samples appears in the lower-left of the STD-NEDDI space (low STD, low NEDDI), which would artificially flatten the boundary if included. These samples are retained in the overall validation but do not drive the global QC threshold.

Consistent with the original manuscript, we fitted two independent boundaries for large and small CNVs:

Large CNVs NEDDI = 2.927 - 16.956 × STD + 25.243 × STD²

Small CNVs NEDDI = 1.115 - 6.319 × STD + 9.701 × STD²

For large CNVs, calibration selected p = 0.48, yielding train specificity = 96.2% and train sensitivity = 83.6%. For small CNVs, p = 0.34 was selected, yielding train specificity = 100.0% and train sensitivity = 83.3%.

**Cross validation**

To assess model stability, we performed 5-fold stratified cross-validation on each training set. Because fail samples are sparse, specificity estimates per fold are noisy; therefore, threshold calibration was performed on the full training set rather than inside individual CV folds. The base model (p = 0.5) achieved CV AUC of 0.933 ± 0.060 for large and 0.955 ± 0.020 for small, confirming robust discriminant power.

In addition, we performed 1000 bootstrap resampling on the training set and refitted the model each time. For the large CNV QC boundary (NEDDI = a + b×STD + c×STD²), the bootstrap means (±SD) of the base-model (p = 0.5) coefficients were a = 3.326 ± 1.217, b = -19.328 ± 7.310, c = 28.780 ± 10.955. For the small CNV QC boundary, a = 1.107 ± 0.300, b = -6.230 ± 1.911, c = 9.542 ± 3.025. The coefficients were stable in sign across all bootstrap iterations and all CV folds, confirming that the qualitative U-shape is robust.

**Portability**

To demonstrate this portability, we conducted two independent validation experiments. In a cross-probe validation (Fig. 1B), we tested the large CNV boundary on 309 samples profiled with xGen (n = 197) and NEXome (n = 112) kits on the same MGISEQ-T7 platform; for each kit, NEDDI was recomputed using the kit-specific cohort median. Both kits aligned with the same quadratic boundary (304 Detectable below, 5 Undetectable above). In a cross-platform validation (Fig. S3), we tested 145 NEXome-captured samples sequenced on MGISEQ-T7 (n = 112) and GeneMind SURFSeq 5000 (n = 33); NEDDI was recomputed using each platform's cohort median. The boundary correctly stratified quality without adjustment (138 Detectable below, 7 Undetectable above).

These cross-probe and cross-platform results confirm that the quadratic boundary coefficients are portable across capture kits and sequencing platforms. The only requirements are that NEDDI is computed from a locally assembled reference cohort median and that experimental conditions remain consistent between the reference cohort and production samples. For implementation, laboratories should first collect 20–30 samples processed under identical conditions to serve as the reference cohort, then compute STD and NEDDI following Supplementary Methods section 1 using the local cohort median as the normalization baseline. The published boundary equations can be applied directly without refitting. We recommend validating on ≥ 10 local samples with known CNV outcomes before deployment, and refreshing the reference cohort annually or after any major protocol change.

**3 WES data calling**

**CNV detection**

A customized workflow based on CNVkit was employed for CNV detection^1^, following recommended protocols with a reference target bin set configured at a maximum size of 240 base pairs. To establish a robust CNV baseline, an iterative training approach was implemented using a randomly selected sample subset. In each iteration, CNVs larger than 5 megabases (Mb) were excluded from the training dataset. This process continued until fewer than 5% of the training samples required exclusion, ensuring at least 20 samples remained in the final baseline set.

Given the sequencing of samples across two platforms (MGISEQ-2000 and MGISEQ-T7), independent baseline models were trained for each platform and applied to platform-specific datasets. CNV calling utilized circular binary segmentation (CBS) and a 3-state hidden Markov model (HMM-germline) to generate a comprehensive set of CNV predictions.

To enhance exon-level CNV detection accuracy, sex-specific CNV baselines were established for each sequencing batch. A Z-score outlier detection algorithm was applied to refine CNV calls, with outliers defined as exons with Z-scores > +3 or < -3. These outlier regions were flagged for orthogonal validation to minimize false positives.

**Mosaicism detection**

Mosaic CNVs were detected using the mrmosaic workflow (https://github.com/asifrim/mrmosaic)^2^ with detected regions cross-validated via B.Allele.Freq (BAF) analysis. For male X chromosomes, mosaic regions were classified based on BAF density thresholds between 0.3 and 0.7. For autosomes and female X chromosomes, regions were flagged as mosaic if their peak BAF densities exceeded 0.6 or fell below 0.4. This dual-modal validation approach minimized false positives and ensured robust mosaicism classification.

**CNV refinement and filtration**

A four-tiered CNV refinement pipeline was implemented to prioritize clinically actionable variants and exclude likely benign findings:

(1) Annotation and class exclusion

Detected CNVs were annotated using AnnotSV^3^, with variants classified as ACMG Classes 1 (benign) or 2 (likely benign) excluded from further analysis.

(2) Redundancy Removal

For overlapping CNVs (> 30% reciprocal-overlap threshold), the highest-ranked ACMG classification determined retention priority.

(3) Population Frequency Filtering

A CNV frequency database was established using a 70% reciprocal-overlap threshold to identify recurrent variants. Variants exceeding 0.01 minor allele frequency in this database were excluded as common polymorphisms.

(4) Expert manual review

For candidate CNVs, manual inspection was performed using intra-batch comparison of normalized depth signals. CNV signals supported by fewer than 1 in 20 neighboring samples were flagged as reliable, while broader batch-wide patterns were flagged for cautious interpretation or technical artifact.

**Synthetic WES data analysis**

For this simulation study, we selected 16 high-quality WES samples (SY01-SY16), each with a Fold-80 value below 1.65. These samples collectively represented a diverse set of CMA-confirmed pathogenic CNVs, including 7 large deletions, 1 large duplication, 3 large mosaic duplications, 4 small deletions, and 1 small duplication. To simulate degraded input, we also included four independent low-quality samples (SY17–SY20) with diverse failure modes, including moderate capture degradation (SY17, Fold-80 = 1.86), GC bias (SY18, GC = 0.555), severe capture inefficiency (SY19, Fold-80 = 5.70), and elevated duplication (SY20, duplication rate = 11.3%) (Table S2). These four low-quality samples and a high-quality sample SY21 (Fold-80 = 1.48) were blended with the 16 baseline samples (SY01–SY16) to generate synthetic mixtures with Fold-80 values ranging from 1.45 to 5.70. These experiments showed that the original CNV‑positive signal was retained only when the high‑quality proportion exceeded 50%, and mixtures below that threshold were excluded from model training.

The total sequencing data size for each synthetic sample was fixed at 9 Gb. Reads were randomly sampled from the raw FASTQ files of both high- and low-quality samples using seqtk (https://github.com/lh3/seqtk) based on predefined synthetic proportions. The sampled reads were then merged and processed through the standard CNV detection pipeline described above. Finally, CNV calls were compiled and compared against CMA validation results to assess detection accuracy under different quality conditions.

**Regions of homozygosity (ROH) and uniparental disomy (UPD) analysis**

ROH were identified using AutoMap^4^, with a minimum size threshold of 5 megabases (Mb). The trio exome sequencing data were analyzed for UPD using UPDio^5^. Detected ROH and UPD regions were annotated using multiple databases to assess associations with imprinted genes (as defined in the Geneimprint database: https://www.geneimprint.com/site/genes-by-species), molecular alterations, or parent-of-origin effect syndromes^6,7^. Only clinically significant findings were retained, with prioritization criteria including: (1) ROH exceeding 10 Mb in size; (2) UPD involving chromosomes with known imprinting effect (chromosomes 6, 7, 11, 14, 15, and 20). These prioritized regions underwent downstream analysis and clinical reporting.

**4 Variants validation**

**Multiplex ligation‑dependent probe amplification (MLPA)**

MLPA was conducted using SALSA MLPA Probe mixes P034-B2/035-B1 DMD, P060-B2 SMA, and P046-D1 TSC2 (MRC-Holland, Amsterdam, the Netherlands) following the manufacturer’s protocols. In brief, genomic DNA was diluted to 30 ng/µL, and 5 µL was dispensed into each reaction tube. Samples were denatured at 98℃ for 5 min, followed by cooling to 25℃. Subsequently, 1.5 µL of probe mix and 1.5 µL of MLPA buffer were added, incubated at 95℃ for 1 min, and hybridized overnight at 60℃ for 16 h.

Ligation was initiated by reducing the temperature to 54℃, followed by the addition of 32 μL ligase mixture and 1 μL of Ligase-65. Samples were incubated at 54℃ for 15 min, heat-inactivated at 98℃ for 5 min, and cooled to 20°C. Post-ligation, 10μL of PCR reaction mix was added and amplification was performed for 35 cycles (95℃ for 30 s, 60℃ for 30 s, and 72℃ for 60 s). Followed by a final extension at 72℃ for 20 min and storage at 15℃.

PCR products were analyzed via capillary electrophoresis on an ABI 3500xL Genetic Analyzer. Data were processed using Coffalyser.NET v1.0 software, and relative copy number was calculated according to the SALSA® MLPA® Probemixes Product Description.

**Gap-polymerase chain reaction (gap-PCR)**

Common α-thalassemia mutations were detected using gap-polymerase chain reaction (gap-PCR) with kits from Yaneng Biosciences (China). The targeted mutations included --^SEA^, -α^3.7^, and -α^4.2^. All experimental procedures were performed in strict accordance with the protocols of the manufacturers.

**Quantitative PCR (qPCR)**

Based on WES-detected CNV boundaries, a pair of specific qPCR primers was designed within each target CNV region, with the RPP30 gene (coding Ribonuclease P/MRP 30-KD subunit) serving as an internal reference. The reaction mix (Accurate Biology) consisted of 10 μL SYBR Green Pro Taq HS mix, 0.6 μL each of forward and reverse primers, 0.2 μL ROX dye, 1 μL DNA template, and ddH_2_O to a final volume of 20 μL. Amplification was conducted on an Applied Biosystems 7500 Real-Time PCR System with pre-denaturation at 95℃ for 2 min, followed by 45 cycles of denaturation at 95℃ for 15 s, annealing at 56℃ for 20 s, and extension at 65℃ for 40 s. Target region copy numbers were determined via relative quantification using Ct values.

**Whole genome sequencing (WGS)**

Genomic DNA was extracted from peripheral blood, amniotic fluid, and umbilical cord blood samples. Extracted DNA was fragmented to 300-500 bp using a Covaris M220 ultrasonicator, followed by library construction using standardized protocols. Paired-end sequencing (150 bp read lengths) was performed on MGISEQ-T7 platforms.

Raw sequencing reads were preprocessed using fastp v0.23.2 to remove low-quality reads and adapter sequences^8^. Cleaned reads were aligned to the human reference genome (G1Kv37) using Sentieon Genomics tools 202308^9^. BAM files were sorted with SAMtools 1.9^10^, and duplicate reads were marked using Picard Tools 2.19.2 (https://broadinstitute.github.io/picard).

For variant analysis, single nucleotide variants (SNVs) were identified using Sentieon Genomics tools 202308^9^. Copy number variations (CNVs) were detected using a customized pipeline that integrated CNVkit v0.9.9 and Delly v0.8.1^1,11^. CNV breakpoints were manually validated in Integrative Genomic Viewer (IGV) v2.16.2^12^, with the "Show soft-clipped bases" option enabled to leverage soft-clipped reads for precise breakpoint delineation. Regions of homozygosity (ROH) were analyzed using Automap^4^, with the "minimal depth for variants" parameter set to 15.

**References**

1.	Talevich E, Shain AH, Botton T, Bastian BC. CNVkit: Genome-Wide Copy Number Detection and Visualization from Targeted DNA Sequencing. *PLoS Comput Biol*. 2016;12(4):e1004873. doi:10.1371/journal.pcbi.1004873

2.	King DA, Sifrim A, Fitzgerald TW, et al. Detection of structural mosaicism from targeted and whole-genome sequencing data. *Genome Res*. 2017;27(10):1704-1714. doi:10.1101/gr.212373.116

3.	Geoffroy V, Herenger Y, Kress A, et al. AnnotSV: an integrated tool for structural variations annotation. *Bioinforma Oxf Engl*. 2018;34(20):3572-3574. doi:10.1093/bioinformatics/bty304

4.	Quinodoz M, Peter VG, Bedoni N, et al. AutoMap is a high performance homozygosity mapping tool using next-generation sequencing data. *Nat Commun*. 2021;12(1):518. doi:10.1038/s41467-020-20584-4

5.	King DA, Fitzgerald TW, Miller R, et al. A novel method for detecting uniparental disomy from trio genotypes identifies a significant excess in children with developmental disorders. *Genome Res*. 2014;24(4):673-687. doi:10.1101/gr.160465.113

6.	Soellner L, Begemann M, Mackay DJG, et al. Recent Advances in Imprinting Disorders. *Clin Genet*. 2017;91(1):3-13. doi:10.1111/cge.12827

7.	Morison IM, Ramsay JP, Spencer HG. A census of mammalian imprinting. *Trends Genet TIG*. 2005;21(8):457-465. doi:10.1016/j.tig.2005.06.008

8.	Chen S, Zhou Y, Chen Y, Gu J. fastp: an ultra-fast all-in-one FASTQ preprocessor. *Bioinforma Oxf Engl*. 2018;34(17):i884-i890. doi:10.1093/bioinformatics/bty560

9.	Freed D, Aldana R, Weber JA, Edwards JS. The Sentieon Genomics Tools - A fast and accurate solution to variant calling from next-generation sequence data. *openRxiv*. Preprint posted online March 10, 2017. doi:10.1101/115717

10.	Danecek P, Bonfield JK, Liddle J, et al. Twelve years of SAMtools and BCFtools. *GigaScience*. 2021;10(2):giab008. doi:10.1093/gigascience/giab008

11.	Rausch T, Zichner T, Schlattl A, Stütz AM, Benes V, Korbel JO. DELLY: structural variant discovery by integrated paired-end and split-read analysis. *Bioinforma Oxf Engl*. 2012;28(18):i333-i339. doi:10.1093/bioinformatics/bts378

12.	Thorvaldsdóttir H, Robinson JT, Mesirov JP. Integrative Genomics Viewer (IGV): high-performance genomics data visualization and exploration. *Brief Bioinform*. 2013;14(2):178-192. doi:10.1093/bib/bbs017

Supplementary Results

**1 Sensitivity analyses**

Sensitivity analyses using alternative reciprocal-overlap thresholds showed that CNV concordance between WES and CMA was 98.51% (199/202) at 30% overlap and 85.15% (172/202) at 70% overlap. These findings indicate that segment-level concordance is sensitive to breakpoint resolution differences between WES and CMA, particularly when CNVs extend into non-captured intergenic regions. Importantly, all six discordant cases under the 50% criterion remained concordant at the gene level, as both methods identified the same clinically relevant pathogenic gene or critical exonic region.

**2 Expanded QC validation**

The QC framework was further evaluated in an expanded cohort of 500 CMA-confirmed clinical samples (Table S4). Among these, 296 samples were negative for P/LP CNVs, 178 carried only large CNVs, 25 carried only small CNVs, and one sample (CW1234) carried both large and small pathogenic CNVs. Separate 2 × 2 contingency tables were constructed for large- and small-CNV QC, and the resulting sensitivity, false-positive rate, and negative predictive value are summarized in Table S10A and Table S10B.

**Table S10A. 2 × 2 contingency table for large-CNV QC**

| Large-CNV QC | Samples Carried P/LP Large CNVs | Negative Samples | Total |
| --- | --- | --- | --- |
| PASS | 173 | 288 | 461 |
| FAIL | 6 | 33 | 39 |
| Total | 179 | 321 | 500 |

**Performance metrics**

Sensitivity = 173/179 = 96.6%

FPR = 33/321 = 10.3% [95% CI: 7.4%–14.1%]

NPV = 33/39 = 84.6% [95% CI: 70.3%–92.8%]

**Table S10B. 2 × 2 contingency table for small-CNV QC**

| Small-CNV QC | Samples Carried P/LP Small CNVs | Negative Samples | Total |
| --- | --- | --- | --- |
| PASS | 25 | 361 | 386 |
| FAIL | 1 | 113 | 114 |
| Total | 26 | 474 | 500 |

**Performance metrics**

Sensitivity = 25/26 = 96.2%

FPR = 113/474 = 23.8% [95% CI: 20.2%–27.9%]

NPV = 113/114 = 99.1% [95% CI: 95.2%–99.8%]

The expanded cohort provides stable NPV estimates for both size classes. For small CNVs, the NPV of 99.1% with a narrow 95% confidence interval of 95.2%-99.8% indicates that a small-QC-fail result is strongly associated with the absence of P/LP small CNVs in this validation cohort. For large CNVs, the NPV of 84.6% indicates that the majority of flagged samples are true negatives. The remaining 15.4% represents missed large CNVs, which is an acceptable trade-off given the clinical priority of preventing poor-quality samples from entering downstream analysis. The FPR of 10.3% for large CNVs indicates that approximately one in ten true-negative samples is flagged for re-sequencing; this rate is comparable to other clinical NGS QC frameworks and reflects the inherent conservatism of the calibrated boundary.

**3 Underlying causes of false-positive CNVs**

The three false-positive CNVs, shown in Table S7, were all small exon-level deletions (50 bp–8.9 kb) detected by WES but not corroborated by orthogonal methods: (1) CW0606: an 8,980 bp heterozygous deletion at 10q23.33 (chr10:94,381,141–94,390,121) spanning exons 10–12 of *KIF11*; (2) CW1807: an 821 bp heterozygous deletion at 22q12.2 (chr22:31,329,419–31,330,240) spanning exons 20–21 of *MORC2*; and (3) CW2634: a 50 bp homozygous deletion at 7q21.11 (chr7:81,642,776–81,642,826) spanning exon 14 of *CACNA2D1*.

These calls reflect distinct technical artifacts inherent to WES-based read-depth analysis. For CW0606, the *KIF11* region showed ~15% lower depth than the intra-batch reference mean, causing a batch-effect–driven drift below the deletion threshold after normalization. For CW1807, the log₂ratio of −0.623141 was substantially weaker than the expected −1.0 for a true heterozygous deletion; this marginal signal, combined with its 821 bp two-exon span, indicates local hybridization noise rather than a genuine loss. For CW2634, the 50 bp exon 14 had virtually no aligned reads, likely due to a probe-design blind spot or homology-driven mapping loss (*CACNA2D1* belongs to a paralogous gene family), yielding a false homozygous deletion. Collectively, these false positives arose from batch bias, sub-threshold signal fluctuation, and capture/mapping null zones—well-documented failure modes for small intragenic CNV detection by WES.
