# Supplementary material for: Systematic evaluation of whole exome sequencing for copy number variation detection in prenatal diagnosis
Source: Clin Transl Med. 2026 Sep 8;16(9):e70761. doi: 10.1002/ctm2.70761 (PMC13554711; doi:10.1002/ctm2.70761)
Supplement: Supplementary file 4 [file CTM2-16-e70761-s005.docx]

**Systematic Evaluation of Whole Exome Sequencing for Copy Number Variation Detection in Prenatal Diagnosis**

**Running Title**

**WES Matches CMA for Prenatal CNVs**

Lihua Yu^1,2,3,4†^, Rong Hu^1,2,3,4†^, Xingwang Wang^1,2,3,4†^, Yongchu Liu^5†^, Haowen Tan^6^, Haoyu Dou^5^, Yi Kong^7^, Jian Lu^1,2,3,4^, Hongke Ding^1,2,3,4^, Yimo Zeng^1,2,3,4^, Cuize Yao^1,2,3,4^, Li'ai Pan^1,2,3,4^, Shuqi Chen^1,2,3,4^, Yan Zhang^1,2,3,4*^, Aihua Yin^1,2,3,4*^

^1^ Medical Genetic Center, Guangdong Women and Children Hospital, Guangzhou, 511442, China

^2^Medical Genetic Center, Women and Children's Hospital, Southern University of Science and Technology, Guangzhou, 511442, China

^3^Guangzhou Key Laboratory of Prenatal Screening and Prenatal Diagnosis, Guangzhou, 511442, China

^4^Maternal and Children Metabolic-Genetic Key Laboratory, Guangzhou, 511442, China

^5^Aegicare (Shenzhen) Technology Co., Ltd., Shenzhen, 518063, China

^6^SynerGene Education, Hejun College, Huichang, 342600, China

^7^Guangzhou Medical University, Guangzhou, 510000, China

**†Lihua Yu, Rong Hu, Xingwang Wang, and Yongchu Liu contributed equally to this work.**

***Correspondence Author**

Yan Zhang, Medical Genetic Center, Guangdong Women and Children Hospital, 521 Xingnan St, Guangzhou, Guangdong 511442, China

Email: zhangyan1981_2003@aliyun.com

Aihua Yin, Medical Genetic Center, Guangdong Women and Children Hospital, 521 Xingnan St, Guangzhou, Guangdong 511442, China

Email: yinaiwa@vip.126.com

**Acknowledgments**

This work was supported by the National Key Research and Development Project (grant number 2024YFC2707001 to Y.Zhang), the National Nature Science Foundation of China (grant number 82171856 to A.Y.) and Guangdong Joint Research Fund (grant number 2022A1515220097 to Y.Zhang).

**Abstract**

**Background**

Chromosomal microarray analysis (CMA) remains the gold standard for detecting prenatal copy number variations (CNVs), while whole exome sequencing (WES) has emerged as a promising alternative with concurrent capabilities for detecting single nucleotide variants (SNVs) and small insertions/deletions (indels). However, systematic evaluation of WES-based CNV detection in prenatal diagnosis remains limited, particularly regarding quality control (QC) standardization and diagnostic concordance with CMA.

**Methods**

We conducted a retrospective cohort study of 3,426 families comprising 3,529 fetuses/probands who underwent parallel CMA and WES. To optimize WES-based CNV calling, we established novel QC metrics—Standard Deviation and Normalized Exon Depth Deviation Index—using simulated datasets.

**Results**

WES demonstrated high concordance with CMA: CNVs (97.03%), mosaicisms (95.83%), and regions of homozygosity (93.22%). Notably, WES identified 18 additional reportable small CNVs (111 bp-89 kb) missed by CMA, including exon-level deletions in *SMN1* and duplications in *DMD*, and resolved compound pathogenic variants (CNVs+SNVs/indels) in four recessive disorder cases. All additional CNVs were validated (overall positive predictive value = 98.65%). Trio-WES showed that 72.58% (90/124) of CNVs classified as variants of uncertain significance were inherited, reducing clinical reporting ambiguity compared with CMA alone.

**Conclusions**

Following fixed QC validation, WES demonstrated diagnostic equivalence to CMA for prenatal CNV detection, excelling in small intragenic variant identification and integrating SNV/CNV analysis. These findings support WES as a robust first-line alternative to CMA for comprehensive genetic evaluation.

**Keywords:** Whole exome sequencing, Chromosomal microarray, Quality control, Prenatal diagnosis

**Introduction**

Copy number variation (CNV), which involves the deletion or duplication of DNA fragments in the genome, is a significant cause of human genetic diseases. Currently, over 300 CNV-related genetic syndromes are known, mainly manifested as developmental delay, abnormal behavior, and organ malformation, etc^1,2^. CNV is also associated with thousands of monogenic genetic diseases like α-thalassemia. In China, about 0.30-6.81 per 10,000 births are affected by CNV-related genetic diseases^3^. Current prenatal genetic testing for fetal structural anomalies often follows a sequential tiered workflow (karyotyping → CMA → WES), which is time-consuming and costly, often leading to delayed comprehensive diagnosis.

Chromosomal microarray analysis (CMA) is widely recognized as the gold standard method for detecting CNVs. It achieves a 6% to 20% diagnostic rate in patients with developmental disorders, congenital anomalies, or fetuses with ultrasound abnormalities^4,5^, securing its position as the first-line test for these conditions^4,6^. However, CMA’s inability to detect single nucleotide variants (SNVs) and small insertions/deletions (indels) simultaneously requires sequential testing strategies, prolonging diagnosis and increasing costs, especially in time-sensitive scenarios like prenatal diagnosis.

Whole exome sequencing (WES), particularly in trio-based analysis, has emerged as a viable alternative. It can detect both CNVs and SNVs/indels. Recent studies have shown that in prenatal cases with negative CMA results, WES can achieve an additional 31% diagnostic rate^7^, and integrated SNV and CNV analysis boosts the diagnostic rate to 46.1%, 13.9% higher than SNV-only analysis^8^. Regions of homozygosity (ROH) represent a category of clinically observable genomic variations that are often associated with consanguineous marriage and uniparental disomy (UPD) and may therefore be linked to specific genetic diseases. Accurate risk assessment requires integration of pedigree information and SNVs, as results from CMA alone cannot accurately evaluate risks.

Despite its strength in detecting SNVs, WES faces challenges in precisely identifying gene-level CNVs due to the heterogeneous exome capture reactions. Computational methods, such as singular value decomposition normalization and ExomeDepth algorithm have been developed^9,10^, but CNV detection reliability is still affected by sequencing depth, sample integrity and data processing. Thus, strict quality control (QC) is essential.

There is ongoing controversy about the reliability of WES-based CNV detection^10–12^. Some studies support WES as a first-tier diagnostic test for neurodevelopmental disorders, while others advocate CMA’s primacy in structural variant detection, particularly in prenatal diagnosis^13,14^. Moreover, limited systematic evaluations compare WES and CMA in CNV detection, and the lack of standardized QC protocols and guidelines hinders WES's clinical adoption as a substitute for CMA.

To fill these gaps, we designed a comprehensive investigation with two objectives: (1) develop robust QC metrics for WES-derived CNV analysis; (2) systematically assess the performance of trio-WES in CNV detection by comparing it with CMA across multiple validated metrics. This study aims to provide empirical evidence supporting WES as a unified first-line genetic diagnostic strategy and to offer practical recommendations for its standardized clinical implementation.

**Materials and Methods:**

**1 Materials**

This study enrolled 3,426 families (3,529 fetuses/probands) who underwent both CMA and WES at the Medical Genetic Center of Guangdong Women and Children Hospital between July 2020 and December 2023. Participants were referred due to congenital anomalies, fetal structural abnormalities identified by ultrasound, or adverse pregnancy history (Table S1). A total of 10,323 individuals underwent WES, with family structures categorized as follows:

29 singleton WES families: Single proband with no parental sequencing.

12 dual-WES families: 11 parent-offspring pairs and 1 sibling pair.

3,270 trio-WES families: 3,268 parent-offspring trios and 2 maternal-twin trios.

115 quad-WES families: 101 twin-parent quadruplets, 13 fetus-proband-parent quadruplets, and 1 fetus-parent quadruplet from two consecutive pregnancies.

The sample types from the 3,529 fetuses/probands were collected as follows:

**Postnatal** samples: 88 peripheral blood samples were collected.

Fetal samples: 3,441 specimens included 249 chorionic villi, 3,098 amniotic fluid, 88 umbilical cord blood, and 6 fetal tissues. In 46 cases, sample types differed between CMA and WES due to insufficient sample quantity or post-termination sampling.

**2 Sequencing and Data Processing**

Genomic DNA was extracted from peripheral blood, amniotic fluid, and umbilical cord blood samples. Using an ultrasonicator (Covaris, US), the DNA was sheared into fragments of 150—300 bp . Standard protocols were then applied for DNA library construction, and target enrichment was achieved using the xGen® Exome Research Panel v1.0 (Integrated DNA Technologies, US). Depending on the recruitment period, the enriched libraries were sequenced using either the MGISEQ-2000 or MGISEQ-T7 platforms (BGI, China) with mean sequencing depths of 100× across target exons, employing paired-end 150-base-pair reads to optimize coverage and data quality.

Raw sequence data were processed through an in-house bioinformatics pipeline integrating publicly available tools. Initially, fastp^15^ was used to remove low-quality reads and adapter sequences. The cleaned reads were aligned to the human reference genome (build G1Kv37) via BWA-MEM^16^, and the generated BAM files were sorted with SAMtools^17^. Picard Tools (https://broadinstitute.github.io/picard/) was employed to identify and mark duplicated reads. For CNV detection, a customized CNVkit-based workflow was implemented^18^. Preliminary candidate CNVs comprised positive segmentation results from either circular binary segmentation (CBS) or a three-state hidden Markov model (HMM)-germline algorithm. Mosaic CNVs were detected using the mrmosaic workflow (<https://github.com/asifrim/mrmosaic>). AutoMap was utilized to identify ROH^19^, with a minimum ROH size set at 5 Mb. Trio-WES data were analyzed for UPD using UPDio^20^. Detailed procedures for detecting CNV, ROH, UPD, and mosaics in WES data were provided in the Supplementary Methods.

**3 Quality Control Metrics Investigation of CNV Calling on WES Data**

Simulations were performed to assess the effectiveness of QC metrics for CNV calling across a systematically controlled gradient of data quality. Conventional metrics—including Fold-80 base penalty (Fold-80), capture efficiency, duplication rate, GC content, and 10× coverage—were evaluated alongside two novel indicators, Standard Deviation (STD) and Normalized Exon Depth Deviation Index (NEDDI), which measure within-sample depth heterogeneity and between-sample depth consistency, respectively.

In this study, CNVs were classified into two size categories: large CNVs, defined as deletions ≥ 100 kb or duplications ≥ 500 kb; and small CNVs, defined as deletions < 100 kb or duplications < 500 kb. Synthetic WES datasets were engineered to mimic clinical data quality heterogeneity by systematically blending high-quality (Fold-80 ≤ 1.65) and low-quality (Fold-80 > 1.85/Dup Rate > 10%) samples in 10% increments (10–90% low-quality content) (Table S2). The high-quality dataset comprised 16 CNV-positive samples including large and small events, each visually validated to exclude false positives. The simulation framework incorporated four independent QC-failed samples representing distinct low-quality patterns, together with high-quality reference samples. These four QC‑failed samples spanned the three most common failure modes in clinical sequencing: capture inefficiency, GC bias, and elevated duplication (Table S2). This generated 720 synthetic mixtures (Table S3). All synthetic mixtures were down-sampled to 9 Gb and processed through the in-house CNV calling pipeline. Two quadratic decision boundaries were then independently fitted for the large- and small-CNV simulations using an inverse-class-frequency-weighted polynomial logistic regression with STD, NEDDI, and STD² as predictors, with the decision threshold tuned to maximize specificity. Coefficient stability was confirmed by 5-fold stratified cross-validation and 1000 bootstrap resampling, as detailed in the Supplementary Methods.

CNV detection performance was benchmarked against CMA (ground truth) by calculating F1-scores for the two size categories using multivariate analysis. The F1-score represents a balanced measure of precision and sensitivity, with true positive defined as events exhibiting ≥ 50% reciprocal overlap. Detailed methodologies for dataset construction, metrics computation, and analysis are provided in the Supplementary Methods.

**4 CMA**

Genomic DNA was extracted from chorionic villus, amniotic fluid, or cord blood using the QIAamp DNA Blood Mini kit (Qiagen, Germany). SNP array analysis was performed with CytoScan 750 K Array (Thermo Fisher Scientific, USA), and annotations were generated using Chromosome Analysis Suite software v4.5 (Thermo Fisher Scientific, USA) based on GRCh37/hg19 genome assembly. Initial CNV calling applied size and probe density thresholds: variants ≥ 100 kb spanning ≥ 50 consecutive markers were considered as candidate CNVs. Additionally, ROHs were screened with a threshold of ≥ 5 Mb^21^. Mosaicism was reported at variant proportions > 10%^21,22^. For CMA-derived CNVs, all retained fragments underwent expert review after initial filtering and screening. This ensured comprehensive identification and reporting of pathogenic/likely pathogenic (P/LP) CNVs, with particular attention to deletions approaching 100 kb and duplications of 500 kb.

**5 CNVs Classification**

All retained CNVs underwent systematic evaluation and classification according to the guidelines of the American College of Medical Genetics and Genomics (ACMG)^21^. Interpretation was supported by public databases including:

Database of Genomic Variants (DGV) (<http://projects.tcag.ca/variation/>)

DECIPHER (<http://decipher.sanger.ac.uk/>)

ClinGen Dosage Sensitivity Map (<https://www.clinicalgenome.org>)

Online Mendelian Inheritance in Man (OMIM) (<http://www.omim.org>)

ClinVar (<https://www.ncbi.nlm.nih.gov/clinvar/>)

For concordance analyses, P/LP CNVs, mosaic CNVs, and ROHs were included and evaluated, with concordance defined as ≥ 50% reciprocal overlap at the segment level. CNV nomenclature for both CMA and WES followed the 2024 International System for Human Cytogenomic Nomenclature (ISCN)^23^.

**6 CNVs Validation**

Segment-level concordance between WES and CMA was defined using a predefined reciprocal-overlap threshold of 50%; the same analytical rule was also applied to ROH comparison. For any discordant cases that fall below this threshold, we employed a validation suite, including multiplex ligation-dependent probe amplification (MLPA), gap-polymerase chain reaction (gap-PCR), quantitative PCR (qPCR), and whole genome sequencing (WGS). Detailed protocols for each validation approach are documented in Supplementary Methods.

**Results**

**1 Quality Control Metrics for CNV Calling on WES Data**

Among all evaluated QC measures, STD and NEDDI emerged as the most reliable predictors of CNV calling accuracy, exhibiting the highest Pearson correlations with F1-score in our simulation study (Fig. S1).

In the controlled dilution experiments, large CNVs remained consistently detectable in mixtures containing up to 40% low-quality input. However, once the proportion of low-quality reads exceeded this threshold, detection reliability began to decline. Specifically, the average overlap rate dropped below 26%, and 240 out of 320 samples (75.0%) failed to detect clinically validated CNV segments. In contrast, small CNVs were highly sensitive to data quality degradation and were entirely undetected in mixtures with 40% or more low-quality input (Table S3). These findings underscore the markedly different sensitivities of large versus small CNV detection to data quality.

Based on the simulation results for large and small CNVs, we fitted two quadratic decision boundaries in STD–NEDDI space to establish a practical QC framework (Fig. 1A and Supplementary Methods). The fitted boundaries are: for large CNV, NEDDI = 2.927 - 16.956 × STD + 25.243 × STD², for small CNV, NEDDI = 1.115 - 6.319 × STD + 9.701 × STD².

Samples falling above the large-CNV boundary failed to meet the quality requirements for reliable detection of either CNV class and should be flagged for resequencing. Samples below the small-CNV boundary met stringent criteria for both large and small CNV detection. Those positioned between the two boundaries maintained robust detection of large CNVs but showed compromised sensitivity for small events, indicating a need for cautious validation before reporting.

The robustness of this QC framework was further evaluated in a validation cohort of 500 CMA-confirmed clinical samples with independent gold-standard labels (Table S4 and Supplementary Results). For large-CNV QC, sensitivity was 96.6%, the false-positive rate (FPR) was 10.3% (95% CI, 7.4%–14.1%), and the negative predictive value (NPV) was 84.6% (95% CI, 70.3%–92.8%) (Fig. S2 and Supplementary Results). For small-CNV QC, sensitivity was 96.2%, the FPR was 23.8% (95% CI, 20.2%–27.9%), and the NPV was 99.1% (95% CI, 95.2%–99.8%) (Fig. S2 and Supplementary Results). Validation across xGen and NEXome XP capture kits, as well as across MGISEQ-T7 and GeneMind SURFSeq 5000 platforms, yielded identical stratification when NEDDI was normalized to local cohort medians, supporting the transferability of the boundary across kits and platforms (Fig. 1B, Fig. S3). A supplementary scatter plot showing the raw STD–NEDDI points and the fitted decision boundaries is provided in Fig. S3.

**2 The Concordance Rates between CMA and WES Results**

**CNVs Detected by CMA and Their Distribution Patterns**

In the present study, CMA identified a total of 202 P/LP CNVs—comprising 146 deletions and 56 duplications (Table S5). Genomic mapping revealed a genome-wide distribution of these variants (Fig. 2). Notable genomic hotspots accounted for 34.65% (70/202) of all detected CNVs. These included 30 variants in chromosomal region 22q11.21, 14 in 15q11.2, 14 in 17q12, and 12 in 16p11.2. In addition to these prominent hotspots, 125 recurrent CNV loci and 77 rare CNV regions were also characterized.

**Comparison of Detection Concordance between WES and CMA for CNVs and Mosaicisms**

Overall, the concordance rate between CNVs detected by CMA and WES was 97.03% (196/202) (Table 1 and Table S5). For deletions, 141 of 146 cases were concordant between CMA and WES, corresponding to a concordance rate of 96.58%. For duplications, 55 out of 56 cases were consistent, yielding a concordance rate of 98.21%. Six cases (5 deletions and 1 duplication) exhibited segment level discordance between CMA and WES. These discrepancies arose because our predefined criterion required ≥ 50% reciprocal overlap threshold to define concordance (sensitivity analyses using alternative thresholds yielded variable rates; see Supplementary Results). In these six discordant cases, the CNV regions contained relatively few OMIM genes, and WES probe coverage was incomplete in these exonic regions, whereas CMA provided genome-wide SNP probe coverage. Notably, the key pathogenic genes were identified by both methods in all six cases. For instance, in Case No. CW1933, WES identified an approximately 37 kb deletion at 8q24.11(118,811,950-118,849,440), whereas CMA revealed a larger deletion of approximately 265 kb. The CNVs detected by the two methods both covered a crucial haploinsufficient gene, *EXT1*.

Among the mosaic cases identified by CMA, 12 involved aneuploidies and 12 involved segmental CNVs. WES corroborated 23 of these 24 events, corresponding to an overall concordance rate of 95.83% (Table 1 and Table S5). Eleven out of 12 mosaic aneuploid cases were identified by WES, yielding a concordance rate of 91.67%. In one discordant case (Case No. CW2676), CMA analysis of chorionic villus samples revealed a mosaic duplication on chromosome 3 (with a mosaic level of approximately 50%). Following genetic counseling, amniocentesis was performed for WES, which detected no P/LP CNVs. Due to discrepancies between the initial CMA results from chorionic villus sampling and the WES results from amniotic fluid, we conducted repeat CMA on the amniotic fluid sample. The final CMA findings were consistent with the WES results, confirming the absence of P/LP CNVs. This discrepancy most likely reflected confined placental mosaicism rather than insufficient sensitivity of WES. Additionally, all P/LP mosaic deletions (n = 3) and duplications (n = 9) showed full concordance with WES results.

**Detection Comparison of Regions of Homozygosity (ROH)**

In the cases with ROHs, the majority (46/51, 90.20%) harbored fewer than three ROHs, whereas five cases exhibited multiple ROHs (Table 2 and Table S6). Notably, cases with multiple ROHs were strongly associated with consanguineous unions. A total of 46 cases comprising 59 ROHs were analyzed, among which 55 ROHs were corroborated by WES, yielding a concordance rate of 93.22%. Among the four discordant ROHs, one was attributed to the absence of the disease-causing genes in the region and insufficient probe coverage. The remaining three cases were classified as discordant because the ROH segments did not meet the 50% reciprocal overlap threshold.

Subsequent SNV analysis within the confirmed ROH regions identified homozygous P/LP SNVs in four cases (7.84%) (Table S7). These variants involved *PKHD1* (Case No. CW0669), *CASR* (Case No. CW1980), *BBS2* (Case No. CW2863), and *RP1* (Case No. CW0466) (Table S7).

In the 43 cases with concordant ROH, integrated family-based WES analysis identified nine instances of UPD, including four cases of segmental mixed UPD. An additional case of mosaic UPD was also identified (Table S6).

**3 Additional Identifications of Small CNVs in CMA-Negative Cases**

Among 3,274 CMA-negative cases, WES detected 18 additional reportable small CNVs (0.55%; 18/3,274), ranging in size from 111 bp to 89 kb (Table S7), including P/LP CNVs and CNVs of uncertain significance classified according to ACMG criteria. All variants were independently validated using other methods, including MLPA, gap-PCR, WGS, or qPCR (Fig. S4). Among these 18 cases, nine (50.00%) harbored homozygous or compound heterozygous deletions within the α-globin gene cluster. One case **(**Case No. **CW0048)** exhibited a homozygous deletion of *SMN1* exons 7–8, and another **(**Case No. **CW2311)** carried a hemizygous duplication of *DMD* exons 31–39. Specifically, a fetus (Case No. CW1363) presented with cerebellar vermis hypoplasia on prenatal ultrasound, which was subsequently confirmed by magnetic resonance imaging (MRI) revealing a slender vermis and thickened superior cerebellar peduncles. Although CMA was negative, subsequent WES identified an approximately 89 kb homozygous deletion at region 2q13, encompassing *NPHP1*, a gene associated with juvenile nephronophthisis or Joubert syndrome. Reexamination of CMA data revealed a deletion signal in this region, but the probe count was < 50, therefore, the finding did not meet the reporting threshold.

Additionally, WES detected compound pathogenic variants—combinations of CNVs with SNVs or indels—in four cases of recessive disorders (0.12%; 4/3,274). Specifically, a fetus (Case No. CW0593) presenting with spinal anomalies, limb shortening, and joint contractures harbored compound heterozygous variants in *SLC26A2* (NM_000112.4): c.1020_1022delTGT (p.Val341del) and a heterozygous deletion of exons 2–3. Another hydropic fetus (Case No. CW2070) exhibited compound heterozygous variants in *CHRNA1* (NM_000079.4), including a c.292dupA (p.Ile98AsnfsTer17) and a heterozygous deletion of exons 2–4.

**4 Positive Predictive Value (PPV) of WES-Detected CNVs**

In this study, WES identified a total of 223 CNVs (Table 3), with a PPV of 98.65% (220/223). Among these, 202 CNVs were corroborated by CMA, and 18 additional reportable small CNVs were validated by alternative methods (Table S5, Table S7, and Fig. S4). The remaining three were classified as false positives, and were all small exon-level deletions (50 bp – 8.9 kb) most consistent with intra-batch reference bias, local hybridization artifacts, or probe-capture blind spots, as detailed in Supplementary Results. In this paired comparison, WES did not miss the clinically relevant pathogenic genes represented in the CMA-positive CNVs, although minor segment-level discordances were observed in six cases (Table 1).

**Discussion**

Contemporary prenatal diagnostic workflows typically employ a tiered genetic testing approach, sequentially utilizing karyotyping, CMA, and WES^24,25^. Currently, studies in pediatrics systematically evaluated WES as a unified first-line test for CNV detection, demonstrating its analytical equivalence to CMA while addressing critical gaps in QC and diagnostic efficiency^8,26^.

A pivotal advancement of this study lies in the establishment of **STD** and **NEDDI** as robust predictors of CNV calling reliability. These metrics outperformed conventional QC indicators and enabled the derivation of data-driven threshold curves to delineate high-quality from unreliable samples. The derived STD–NEDDI quadratic boundaries provide standardized criteria for data reliability assessment, addressing an important barrier to the clinical implementation of WES‑based CNV analysis by offering a systematic approach for identifying samples suitable for confident interpretation or resequencing.

This QC framework addresses a critical gap in WES-based CNV reporting by providing **standardized criteria for data reliability assessment**^8,26,27^. Crucially, its performance was validated across two capture kits and two sequencing platforms, demonstrating platform-agnostic generalizability.

**To further improve the confirmation rate of the QC scheme on real clinical samples and move closer to complete coverage, two iterative strategies could be considered. First, maintaining consistent experimental conditions can help reduce unexpected fluctuations in QC values, such as NEDDI. Second, expanding the reference pool used to calculate exon-level population medians would make the NEDDI metric more robust, particularly when matching reference samples to the sequencing depth of the test sample.**

**Against** CMA as the gold standard, WES achieved an overall CNV concordance rate of 97.03% (196/202) without missing any CMA-identified positive events. The six discordant cases primarily reflected differences in detection resolution: CMA’s genome-wide SNP probe coverage identified larger CNVs, while WES focused on exonic regions containing pathogenic genes (e.g., EXT1 in Case No. CW1933). Critically, **WES detected 18 small CNVs (111 bp–89 kb) missed by CMA**, including intragenic deletions in SMN1 (spinal muscular atrophy) and duplications in DMD (Duchenne muscular dystrophy), highlighting its unique value in diagnosing monogenic disorders reliant on exon-level resolution^28^. Combined with additional findings, the total PPV for WES-derived CNV was 98.65%.

Moreover, WES demonstrated comparable performance in detecting mosaicism (95.83% concordance) and ROH (93.22% concordance), traditionally considered CMA strengths^29–31^. The integration of SNV and CNV analysis within WES enabled the identification of compound pathogenic variants in recessive disorders (e.g., SLC26A2 and CHRNA1), a capability absent in CMA-only workflows. This multimodal detection reduces diagnostic delays by eliminating the need for sequential testing, a critical advantage in time-sensitive prenatal settings.

By combining CNV and SNV detection in a single assay, WES addresses the limitations of CMA’s sequential testing paradigm. The identification of 18 additional reportable small CNVs by WES in CMA-negative cases further highlighted its value in resolving otherwise occult genetic etiologies. Based on our local cost accounting, compared with a sequential CMA + WES workflow, an integrated WES-based single test was estimated to reduce total testing costs by approximately 30%, while shortening turnaround time from 6–8 weeks to 3 weeks^32^. This efficiency is particularly impactful for high-risk pregnancies, where timely diagnosis informs critical management decisions (e.g., Case No. CW2070, where WES detected a prenatal-onset nephropathy-associated CNV missed by CMA).

Despite these advances, several limitations should be acknowledged. First, the single-center retrospective design may introduce selection bias, and external validation via multicenter studies is essential to confirm generalizability. Second, because WES is primarily exon-focused, its ability to detect deep intronic and other non-coding CNVs remains limited, although targeted probe design for known disease-associated regions (e.g., α-globin gene cluster) can partially mitigate this gap. Third, although the STD–NEDDI framework showed stable performance across the evaluated datasets, its threshold may still be influenced by reference-cohort composition, enrichment design, and sequencing chemistry, requiring local validation before clinical implementation. Fourth, although our simulation incorporated multiple low-quality patterns, it may still not capture the full spectrum of real-world prenatal sequencing failures. Fifth, the high concordance we observed does not establish a zero false‑negative rate for WES‑based CNV calling, as our analysis was anchored to CMA‑positive reference cases. Potential false negatives may arise from GC‑rich regions, repetitive elements, and poorly captured exons; although our QC framework helps mitigate this risk, a systematic evaluation will require large‑scale prospective studies using orthogonal methods. Finally, the interpretation of variants of uncertain significance (VUS) remains challenging, though trio analysis reduced VUS reporting rate by 72.58% (Table S8 and Table S9) via parental inheritance tracking^33^.

Beyond VUS interpretation, we acknowledge that periodic re‑analysis of negative cases in the future, particularly as CNV detection algorithms and reference databases improve, may identify additional CNVs and provide valuable insights into the evolving sensitivity of WES‑based CNV calling.

Although our study focused on prenatal samples, the QC framework is likely applicable to pediatric populations. A similar systematic evaluation in postnatal cohorts would be valuable and is planned for future work. Looking forward, the integration of our QC framework with machine learning algorithms could further optimize CNV calling accuracy, particularly for small CNVs. Prospective studies evaluating WES as a first-line approach may also expand its applicability to early pregnancy screening, aligning with the evolving landscape of precision prenatal medicine.

**Conclusion**

This study establishes WES as a robust alternative to CMA for prenatal CNV detection, supported by a validated QC framework and demonstrated diagnostic equivalence. By enabling concurrent SNV/CNV analysis within a single workflow, WES streamlines genetic diagnostics, enhances diagnostic yield for small and intragenic variants, and addresses the unmet needs of monogenic disorder screening. These findings advocate for WES as a first-line test in prenatal settings, particularly when combined with family-based trio analysis to maximize interpretive accuracy.

**Declarations**

**Ethics Approval and Consent to Participate**

This study was approved by the Guangdong Women and Children Hospital Medical Ethics Committee (Approval Number: 20251014). Informed consent was obtained from all participants. For minors, incapacitated individuals, and prenatal cases, consent was provided by their parents or guardians.

**Conflict of Interest Statement**

Y.L. and H.Dou are employee of Aegicare Technology Co., Ltd. Other authors declare that there are no conflicts of interest.

**Data availability**

The data underlying this article are available in the article and in its online supplementary file.

**Author contributions**

Conceptualization, A.Y., Y.Zhang.; Data curation, L.Y., R.H., X.W., H.Dou, H.Ding, J.L.; Formal analysis, X.W., H.T., H.Dou.; Funding acquisition, A.Y., Y.Zhang.; Supervision, A.Y., Y.Zhang.; Validation, L.Y., Y.K., Y.Zeng, C.Y., L.P., S.C.; Visualization, L.Y., R.H., X.W., Y.L., H. Dou.; Writing - original draft, L.Y., R.H., X.W., Y.L., H.T., H.Dou, Y.Zhang.; Writing - review & editing, All authors.
**Reference**

1.	Rehm HL, Berg JS, Brooks LD, et al. ClinGen--the Clinical Genome Resource. *N Engl J Med*. 2015;372(23):2235-2242. doi:10.1056/NEJMsr1406261

2.	Weise A, Mrasek K, Klein E, et al. Microdeletion and Microduplication Syndromes. *J Histochem Cytochem*. 2012;60(5):346-358. doi:10.1369/0022155412440001

3.	Chen X, Lin D, Ye Y, Zhang X, Chen D. Trends in the prevalence, prenatal diagnosis, and outcomes of births with chromosomal abnormalities: a hospital-based study in Zhejiang Province, China during 2014–2020. *Orphanet J Rare Dis*. 2022;17:446. doi:10.1186/s13023-022-02594-1

4.	Miller DT, Adam MP, Aradhya S, et al. Consensus Statement: Chromosomal Microarray Is a First-Tier Clinical Diagnostic Test for Individuals with Developmental Disabilities or Congenital Anomalies. *The American Journal of Human Genetics*. 2010;86(5):749-764. doi:10.1016/j.ajhg.2010.04.006

5.	Wapner RJ, Martin CL, Levy B, et al. Chromosomal Microarray versus Karyotyping for Prenatal Diagnosis. *N Engl J Med*. 2012;367(23):2175-2184. doi:10.1056/NEJMoa1203382

6.	Ravel JM, Renaud M, Muller J, et al. Clinical utility of periodic reinterpretation of CNVs of uncertain significance: an 8-year retrospective study. *Genome Med*. 2023;15(1):39. doi:10.1186/s13073-023-01191-6

7.	Mellis R, Oprych K, Scotchman E, Hill M, Chitty LS. Diagnostic yield of exome sequencing for prenatal diagnosis of fetal structural anomalies: A systematic review and meta‐analysis. *Prenatal Diagnosis*. 2022;42(6):662-685. doi:10.1002/pd.6115

8.	Lan X, Tang X, Weng W, et al. Diagnostic Utility of Trio-Exome Sequencing for Children With Neurodevelopmental Disorders. *JAMA Netw Open*. 2025;8(3):e251807. doi:10.1001/jamanetworkopen.2025.1807

9.	Krumm N, Sudmant PH, Ko A, et al. Copy number variation detection and genotyping from exome sequence data. *Genome Res*. 2012;22(8):1525-1532. doi:10.1101/gr.138115.112

10.	Rajagopalan R, Murrell JR, Luo M, Conlin LK. A highly sensitive and specific workflow for detecting rare copy-number variants from exome sequencing data. *Genome Med*. 2020;12(1):14. doi:10.1186/s13073-020-0712-0

11.	Babadi M, Fu JM, Lee SK, et al. GATK-gCNV enables the discovery of rare copy number variants from exome sequencing data. *Nat Genet*. 2023;55(9):1589-1597. doi:10.1038/s41588-023-01449-0

12.	Hong CS, Singh LN, Mullikin JC, Biesecker LG. Assessing the reproducibility of exome copy number variations predictions. *Genome Med*. 2016;8(1):82. doi:10.1186/s13073-016-0336-6

13.	Srivastava S, Love-Nichols JA, Dies KA, et al. Meta-analysis and multidisciplinary consensus statement: exome sequencing is a first-tier clinical diagnostic test for individuals with neurodevelopmental disorders. *Genet Med*. 2019;21(11):2413-2421. doi:10.1038/s41436-019-0554-6

14.	Yaron Y, Ofen Glassner V, Mory A, et al. Exome sequencing as first-tier test for fetuses with severe central nervous system structural anomalies. *Ultrasound Obstet Gynecol*. 2022;60(1):59-67. doi:10.1002/uog.24885

15.	Chen S, Zhou Y, Chen Y, Gu J. fastp: an ultra-fast all-in-one FASTQ preprocessor. *Bioinformatics*. 2018;34(17):i884-i890. doi:10.1093/bioinformatics/bty560

16.	Freed D, Aldana R, Weber JA, Edwards JS. The Sentieon Genomics Tools - A fast and accurate solution to variant calling from next-generation sequence data. *openRxiv*. Preprint posted online March 10, 2017. doi:10.1101/115717

17.	Danecek P, Bonfield JK, Liddle J, et al. Twelve years of SAMtools and BCFtools. *Gigascience*. 2021;10(2):giab008. doi:10.1093/gigascience/giab008

18.	Talevich E, Shain AH, Botton T, Bastian BC. CNVkit: Genome-Wide Copy Number Detection and Visualization from Targeted DNA Sequencing. *PLoS Comput Biol*. 2016;12(4):e1004873. doi:10.1371/journal.pcbi.1004873

19.	Quinodoz M, Peter VG, Bedoni N, et al. AutoMap is a high performance homozygosity mapping tool using next-generation sequencing data. *Nat Commun*. 2021;12(1):518. doi:10.1038/s41467-020-20584-4

20.	King DA, Fitzgerald TW, Miller R, et al. A novel method for detecting uniparental disomy from trio genotypes identifies a significant excess in children with developmental disorders. *Genome Res*. 2014;24(4):673-687. doi:10.1101/gr.160465.113

21.	Riggs ER, Andersen EF, Cherry AM, et al. Technical standards for the interpretation and reporting of constitutional copy-number variants: a joint consensus recommendation of the American College of Medical Genetics and Genomics (ACMG) and the Clinical Genome Resource (ClinGen). *Genet Med*. 2020;22(2):245-257. doi:10.1038/s41436-019-0686-8

22.	Hall GK, Mackie FL, Hamilton S, et al. Chromosomal microarray analysis allows prenatal detection of low level mosaic autosomal aneuploidy. *Prenat Diagn*. 2014;34(5):505-507. doi:10.1002/pd.4333

23.	ISCN 2024 - An International System for Human Cytogenomic Nomenclature (2024). *Cytogenet Genome Res*. 2024;164(Suppl 1):1-224. doi:10.1159/000538512

24.	Van den Veyver IB, Chandler N, Wilkins-Haug LE, Wapner RJ, Chitty LS, ISPD Board of Directors. International Society for Prenatal Diagnosis Updated Position Statement on the use of genome-wide sequencing for prenatal diagnosis. *Prenat Diagn*. 2022;42(6):796-803. doi:10.1002/pd.6157

25.	Sparks TN, Dugoff L. How to choose a test for prenatal genetic diagnosis: a practical overview. *Am J Obstet Gynecol*. 2023;228(2):178-186. doi:10.1016/j.ajog.2022.08.039

26.	Hahn E, Dharmadhikari AV, Markowitz AL, et al. Copy number variant analysis improves diagnostic yield in a diverse pediatric exome sequencing cohort. *NPJ Genom Med*. 2025;10(1):16. doi:10.1038/s41525-025-00478-4

27.	Zeng Y, Ding H, Wang X, et al. High positive predictive value of CNVs detected by clinical exome sequencing in suspected genetic diseases. *J Transl Med*. 2024;22(1):644. doi:10.1186/s12967-024-05468-1

28.	Wojcik MH, Lemire G, Berger E, et al. Genome Sequencing for Diagnosing Rare Diseases. *N Engl J Med*. 2024;390(21):1985-1997. doi:10.1056/NEJMoa2314761

29.	Society for Maternal-Fetal Medicine (SMFM). Electronic address: pubs@smfm.org, Dugoff L, Norton ME, Kuller JA. The use of chromosomal microarray for prenatal diagnosis. *Am J Obstet Gynecol*. 2016;215(4):B2-9. doi:10.1016/j.ajog.2016.07.016

30.	Kearney HM, South ST, Wolff DJ, et al. American College of Medical Genetics recommendations for the design and performance expectations for clinical genomic copy number microarrays intended for use in the postnatal setting for detection of constitutional abnormalities. *Genet Med*. 2011;13(7):676-679. doi:10.1097/GIM.0b013e31822272ac

31.	Silva M, de Leeuw N, Mann K, et al. European guidelines for constitutional cytogenomic analysis. *Eur J Hum Genet*. 2019;27(1):1-16. doi:10.1038/s41431-018-0244-x

32.	Gabriel H, Korinth D, Ritthaler M, et al. Trio exome sequencing is highly relevant in prenatal diagnostics. *Prenat Diagn*. 2022;42(7):845-851. doi:10.1002/pd.6081

33.	Lord J, McMullan DJ, Eberhardt RY, et al. Prenatal exome sequencing analysis in fetal structural anomalies detected by ultrasonography (PAGE): a cohort study. *Lancet*. 2019;393(10173):747-757. doi:10.1016/S0140-6736(18)31940-8

Table 1 **Consistency between CMA and WES in Detecting CNVs and Mosaicisms**

| Subgroups |  | **CMA Detected** CNVs | **WES Detected** CNVs  With ≥ 50% reciprocal overlap | **Concordance Rate** (95% CI) |
| --- | --- | --- | --- | --- |
| P/LP CNVs | Deletions | 146 | 141 | 96.58% (92.19%-98.88%) |
|  | Duplications | 56 | 55 | 98.21% (90.45%-99.95%) |
|  | **Total** | **202** | **196^†^** | **97.03%** (93.65%-98.90%) |
| Mosaic | Aneuploidy | 12 | 11**^‡^** | 91.67% (61.52%-99.79%) |
|  | P/LP Deletions | 3 | 3 | 100% (29.24%-100%) |
|  | P/LP Duplications | 9 | 9 | 100% (66.37%-100%) |
|  | **Total** | **24** | **23** | **95.83%** (78.88%-99.89%) |

CI: Confidence Interval; P/LP: Pathogenic/Likely Pathogenic

**^†^** The six cases were classified as non-concordant because they did not meet the predefined reciprocal overlap threshold (≥50%), despite agreement at the clinically relevant gene level.

**^‡^**The discordance in one sample (Case No. CW2676) was attributed to biological heterogeneity between chorionic villi and amniocytes.

Table 2 **Comparison of Consistency in Detection of ROHs**

|  | | CMA | | WES | | **Concordance** Rate |
| --- | --- | --- | --- | --- | --- | --- |
|  |  | ROHs | Cases with ROH | ROHs | Cases with ROH |  |
| ROH | ≤ 3 ROHs/Cases | 59 | 46 | 55**^†^** | 43 | **93.22% (55/59)** |
|  | Mutiple ROHs in one case | / | 5 | / | 5 | / |
|  | Mosaic Uniparental Disomy | 1 | 1 | 1 | 1 | 100% |

**^†^**Among four discordant ROHs, three discordances were attributed to < 50% reciprocal overlap threshold with CMA results; one case lacked capture probes.

Table 3 Validation of CNVs Detected by WES

|  |  | CNVs Detected by WES | Validated CNVs by Other Methods | PPV (95% CI) |
| --- | --- | --- | --- | --- |
| Deletions | ≥ 100 kb | 143 | 143 | 100% (97.45%-100%) |
|  | < 100 kb | 23 | 20 | 86.96% (66.41%-97.22%) |
| Duplications | ≥ 500 kb | 54 | 54 | 100% (93.40%-100%) |
|  | < 500 kb | 3 | 3 | 100% (29.24%-100%) |
|  | **Total** | **223** | **220** | **98.65% (96.12%-99.72%)** |

PPV: Positive Predictive Value; CI: Confidence Interval

**Figures**

**Fig. 1 Evaluating CNV Detection Performance Using F1-Score and QC Metrics.** (a) A scatter plot illustrates the relationship between QC metrics, STD, and NEDDI. Points on the plot represent samples; simulated mixtures with less than 50% high-quality content are shown transparent. A color gradient, from red (high F1-score) to blue (low F1-score), indicates detection accuracy. A dashed boundary distinguishes high-performance samples (F1-score ≥ 0.3) from low-performance ones (F1-score < 0.3). (b) This comparison of QC metric distributions between IDT (circular markers) and NEXome (triangular markers) probe designs reveals that NEXome XP Panel v1.0 samples closely match IDT patterns, underscoring the QC metrics' generalizability across different probe designs.

**Fig. 2 Chromosomal Distribution of CNVs in the Genome Detected by CMA.** The tracks, arranged from the outermost to the innermost circles, are as follows: (1) an ideogram-based representation of chromosomes; (2) CNVs, where red indicates duplications, blue indicates deletions, and the height of the bars represents the frequency of CNVs; and (3) chromosomal regions associated with CNVs.
